# Bone loss is ameliorated by fecal microbiota transplantation through SCFA/GPR41/ IGF1 pathway in sickle cell disease mice

**DOI:** 10.1038/s41598-022-25244-9

**Published:** 2022-11-30

**Authors:** Liping Xiao, Yanjiao Zhou, Suresh Bokoliya, Qingqi Lin, Marja Hurley

**Affiliations:** Department of Medicine MC3023, School of Medicine, UConn Health, 263 Farmington Avenue, Farmington, CT 06030-3105 USA

**Keywords:** Immunology, Diseases

## Abstract

Bone loss is common in sickle cell disease (SCD), but the molecular mechanisms is unclear. Serum insulin-like growth factor 1 (IGF1) was low in SCD subjects and SCD mice. To determine if decreased IGF1 associated with low bone mass in SCD is due to reduced SCFA production by gut microbiota, we performed reciprocal fecal microbiota transplantation (FMT) between healthy control (Ctrl) and SCD mice. uCT and histomorphometry analysis of femur showed decreased bone volume/total volume (BV/TV), trabecular number (Tb.N), osteoblast surface/bone surface (Ob.S/BS), mineralizing surface/ bone surface (MS/BS), inter-label thickness (Ir.L.Th) in SCD mice were significantly improved after receiving Ctrl feces. Bone formation genes *Alp, Col1, Runx2*, and *Dmp1* from SCD mice were significantly decreased and were rescued after FMT from Ctrl feces. Transplantation of Ctrl feces increased the butyrate, valerate, and propionate levels in cecal content of SCD mice. Decreased G-coupled protein receptors 41 and 43 (*GPR41* and *GPR43*) mRNA in tibia and lower IGF1 in bone and serum of SCD mice were partially restored after FMT from Ctrl feces. These data indicate that the healthy gut microbiota of Ctrl mice is protective for SCD bone loss through regulating IGF1 in response to impaired bacterial metabolites SCFAs.

## Introduction

SCD is the most common genetic disorder worldwide^1^. Osteoporosis and low bone mineral density (BMD), or osteopenia, are common complications in both children and adults sickle cell patients^2^. Although osteoarticular complications usually do not lead to mortality, they do result in a significant amount of morbidity, with long-term disability, fracture, and chronic pain^3–7^. Eighty percent of adults sickle cell patients have low BMD. The cause of bone loss in SCD is different from the general population since low BMD in SCD is independent of usual risk factors such as menopausal status, gender, and age^8^. However, the mechanism(s) of bone loss in human SCD has not been thoroughly investigated.

Microbiota are ecological communities of commensal, symbiotic, and pathogenic microorganisms^9^. The intestinal microbiota contains bacteria, fungi, viruses, and archaea^10^. The intestinal bacteria (more than 100 trillion) include approximately 1000 different species from 29 bacterial phyla^10^. The gut microbiota is very important for the host health since it plays roles in degradation of non-digestible polysaccharides, strengthening gut integrity or shaping the intestinal epithelium, harvesting energy, protecting against pathogens, and regulating host immunity^11,12^. Microbiota dysbiosis (imbalanced gut microbiota) is associated with diseases such as inflammatory bowel disease, diabetes, and obesity^13–16^. A recent intestinal microbiome analysis study revealed dysbiosis in SCD in human^17^. Studies from Frenette’s group showed that gut microbiota regulate neutrophil aging that mediates the inflammation-related liver and spleen damage^18^, and regulate psychological stress induced inflammation in SCD mice^19^. We published that depletion of gut microbiota with antibiotics in part ameliorate bone loss in sickle cell disease mice^20^ suggesting a link between gut microbiota and bone loss in SCD.

Through production of specific metabolites or through inducing host response to symbiont-associated molecular patterns microbiota may influence host physiology^21–26^. Short chain fatty acids (SCFAs) are bacterial fermentation products generated primarily in the colon from undigested dietary carbohydrates and have been involved in the host immune system maturation, such as increasing peripheral regulatory T cells in peripheral, protecting infection, modulating energy homeostasis and metabolic rate^22–26^. SCFAs can directly regulate osteoclasts (OCs) and osteoblasts (OBs) function^27^. SCFAs can also regulate insulin-like growth factor 1 (IGF1) production and subsequently affect bone growth^21^. Serum IGF1 was decreased in SCD patients^28^. We reported that the reduced mechanical properties and bone mass in SCD mice were related with reduced IGF1 in serum and bone, and reduced osteoblast terminal differentiation marker gene expression^29^. Although it has already been established that gut microbiome dysbiosis leads to bone loss and that effects playing out in this context are related to SCFAs and altered IGF1^21^, there is no published study on SCFA levels in SCD patients or SCD mice. It is not known if decreased IGF1 in SCD is because of decreased SCFA production via gut microbiota.

The human microbiota has been shown to influence a number of conditions associated with impaired bone quality^30,31^. Given the powerful role of the intestinal microbiota in regulating bone health, we hypothesized that microbial dysbiosis contributes to bone pathogenesis in SCD mice. Specifically, we proposed that healthy balanced gut microbiota community from healthy control (Ctrl) mice confers protection from SCD-related bone loss by changing the production of bone growth factor IGF1 in response to altered bacterial metabolites SCFAs. Here we report novel findings that gut microbiota dysbiosis contributes to bone loss in SCD mice. We further show that gut microbiota dysbiosis causes decreased SCFA production and decreased IGF1 in bone and serum. Fecal microbiota transplantation (FMT) from Ctrl feces to SCD mice prevented SCD-mediated osteoblast dysfunction and bone loss.

## Materials and methods

### Mice

The Townes sickle cell mice and Ctrl mice in a C57BL/6;129 background were obtained from the Jackson Laboratory (Stock number: 013071).Townes sickle cell mouse model is unique and displays major features found in humans with SCD^32^. It has both human α- and β-globin genes knocked into the mouse locus. Crossing sickle cell trait (heterozygous, AS) mice allows the generation of Ctrl (healthy, AA) and SCD (homozygous, SS) littermates. Both male and female mice were used and independent analyses were conducted for each gender. All methods involving animals were performed according to the UConn Health’s regulations and guidelines, and in compliance with the ARRIVE guidelines. The UConn Health Institutional Animal Care and Use Committee approved all protocols involving animals.

### Fecal microbial community analysis

Fecal pellets were collected from 3-month old Ctrl and SCD female mice (18–24 mice/group). Pellets were stored in deep freezer (− 80 °C) until processing for microbiota analysis. Pellets were processed with StrainID Kit (Shoreline Biome) for microbial community profiling. Briefly, DNA was purified from mouse fecal pellets and StrainID amplicons were prepared and pooled for sequencing as per manufacturer’s instructions. Pooled DNA quality (A_260_/A_280_ ratio) and concentration were determined by a Nanodrop 1000 spectrophotometer (Thermo Fisher Scientific, USA). The StrainID amplicon encompasses a ~ 2500 bp region including the 16S rRNA gene, the adjacent internally transcribed spacer region, and part of the 23S gene. SMRTbellTM library prep and sequencing was performed at the University of Delaware on a PacBio Sequel II. Circular consensus reads were mapped to the Athena 16S-23S rRNA database using SBanalyzer.

### Fecal microbiota transplantation (FMT)

FMT was performed from Ctrl to SCD to test if Ctrl microbiota can rescue SCD-like bone phenotypes and from SCD to Ctrl to test for a pathogenic effect of SCD microbiota on Ctrl mice. To prepare RECIPIENT mice for FMT, Ctrl or SCD female mice (3 months old, 10 mice/group) were fasted for 1 h, then mice were given oral gavage of four doses of 200 μl PEG (polyethylene glycol, Macrogol 4000) at 425 g/L at 20-min intervals for bowel cleansing^33,34^ (to remove the majority of their gut flora to enhance FMT efficiency by promoting xenomicrobiota colonization in the intestinal mucosa^33,34^). The efficiency of removing gut flora by laxative PEG was confirmed by RT-qPCR quantifying the abundance of commensal bacteria using the universally conserved 16S rRNA (Supplemental Fig. 1). To prepare DONOR fecal matter, stool samples were collected from donor mice and stored at − 80 °C until processed. The preparation of stool for fecal microbiota transplantation was performed under an anaerobic hood. The stool samples were homogenized with sterile brain–heart infusion (BHI) broth. BHI was pre-reduced by degassing with anaerobic gas mixture N2/H2/CO2 (90%:5%:5%) followed by supplementation of antioxidant 0.1% l-cysteine hydrochloride monohydrate. Further, filtration was performed using a 0.45-micron filter to remove undissolved particulate matter and stool suspensions stored in aliquots for FMT. FMT was performed by oral gavage to recipient mice 4 h after last PEG treatment. Ctrl recipient receiving Ctrl feces and SCD recipient receiving SCD feces are controls. FMT was conducted once per week. Six weeks after FMT, bone samples were harvested. Similar FMT was also performed in male mice.

**Figure 1:**
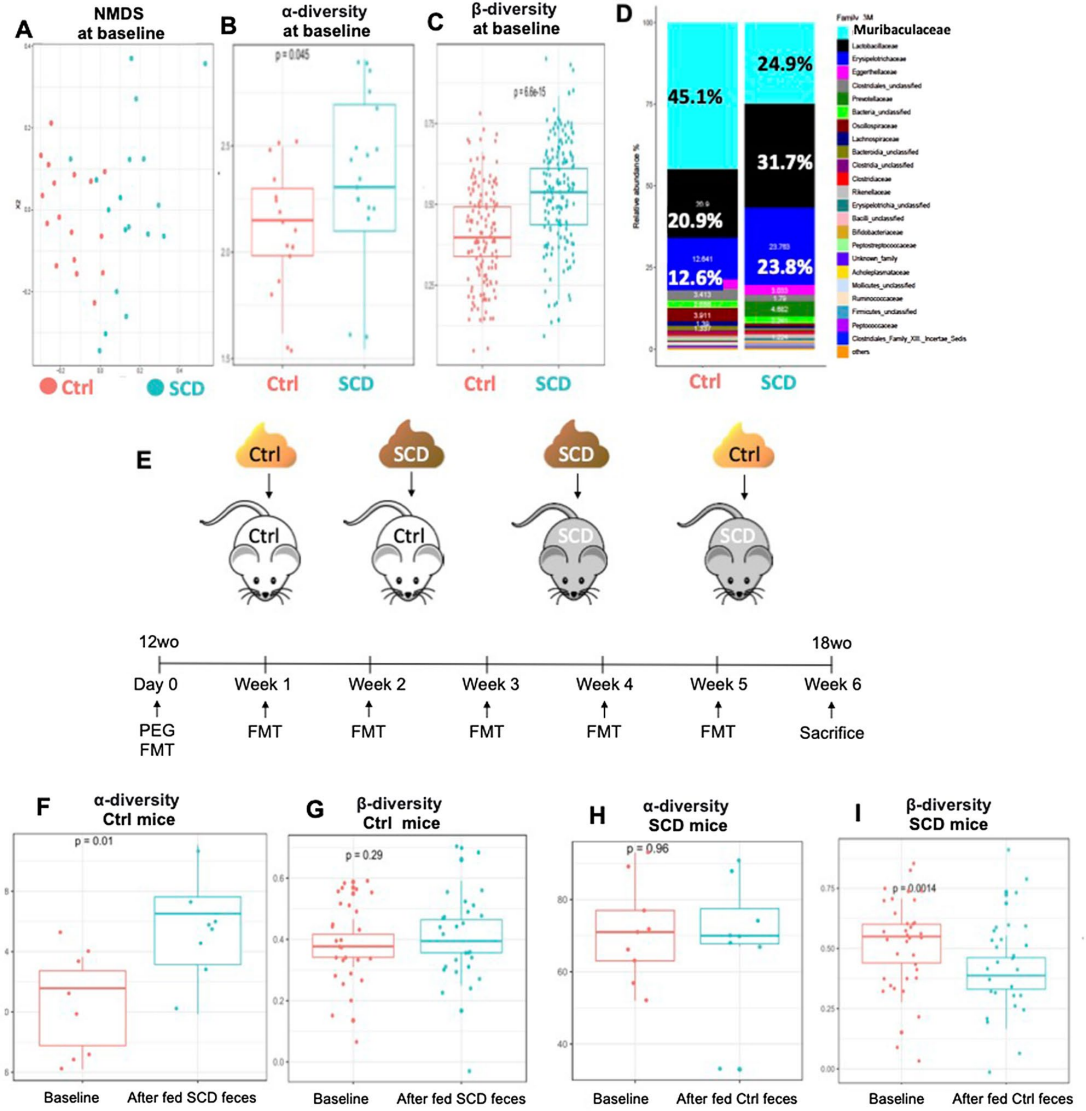
Microbiota composition and reciprocal FMT in Ctrl and SCD mice. (**A**–**D**) Ctrl and SCD mice have distinct microbiota. Feces were collected from 3-month-old female mice. (**A**) NMDS plots of Amplicon Sequence Variants (ASVs) show that samples from the two groups cluster separately, indicating two distinct microbiota communities. n = 17–18 mice/group. (**B**,**C**) Analysis of ASVs shows that (**B**) alpha-diversity (**C**) beta-diversity increases significantly in SCD mice. n = 17–18 mice/group. (**D**) Relative abundance of top 25 bacteria families. Data are mean % over total bacteria. n = 17–18 mice/group. (**E**) Scheme of reciprocal FMT to examine whether healthy Ctrl microbiota can ameliorate SCD- related bone loss and from SCD to Ctrl to examine for a pathogenic effect of SCD microbiota on Ctrl mice. Reciprocal FMT was conducted from Ctrl to SCD to examine whether Ctrl microbiota can ameliorate SCD-related bone loss and from SCD to Ctrl to examine for a pathogenic effect of SCD microbiota on Ctrl mice. To prepare RECIPIENT mice for FMT, Ctrl or SCD female mice (3 months old, 10 mice/group) were fasted for 1 h, then mice were given bowel cleansing with four doses of PEG by oral gavage at 20-min intervals to remove the majority of their gut flora to enhance FMT efficiency. To prepare DONOR fecal matter, one fresh fecal pellet per mouse was collected from age and gender matched donor mice (8 mice/genotype) and the pooled whole fecal matter was dissolved in 2 ml of PBS and given by oral gavage 4 h after last PEG treatment to recipient mice. Ctrl recipient receiving Ctrl feces and SCD recipient receiving SCD feces are controls. FMT was conducted once per week with fresh feces collected from donor mice. Six weeks post FMT, bone samples were collected. Similar FMT was also performed on male mice. wo: weeks old. Analysis of ASVs of (**F**) alpha-diversity and (**G**) beta-diversity in Ctrl mice at baseline and after fed SCD feces; (**H**) alpha-diversity and (**I**) beta-diversity in SCD mice at baseline and after fed Ctrl feces. n = 8–9 mice/group.

### Micro-computed tomography (μCT)

Micro-CT (μCT40; Scanco Medical AG) was conducted on mid-diaphysis of femur for cortical morphometry, and within the metaphyseal region of distal femurs for trabecular morphometry as described in our previous publication^29^. Trabecular morphometric parameters included trabecular volume fraction (BV/TV), trabecular spacing (Tb.Sp), trabecular number (Tb.N), trabecular thickness (Tb.Th). Cortical morphometry included measures of cortical area (Ct.Ar), cortical thickness (Ct.Th), cortical area fraction (Ct.Ar/Tt.Ar), and total area (Tt.Ar)^35^.

### Bone histomorphometry

Mice were injected intraperitoneally with calcein 7 days and xylenol orange 2 days before sacrificing, respectively. Femurs were excised and fixed in 10% formalin. Then femurs were in 30% sucrose dissolved in PBS for overnight and embedded in Cryomatrix. Six- μm longitudinal section of femur were collected. Unstained sections were used for dynamic parameters analysis. Additional femur sections were used for tartrate-resistant acid phosphatase (TRAP) staining. The OsteoMeasure image analysis system (R&M Biometrics) was used for histomorphometry measurement. The terminology and units used are those recommended by the Histomorphometry Nomenclature Committee of the American Society for Bone and Mineral Research^36^. Mineralizing surface/ bone surface (MS/BS), the bone formation rate (BFR)/BS, inter-label thickness (Ir.L.Th), OC surface (Oc.S/BS), and OC number/BS (N.Oc/BS) , and percent OB surface (Ob.S/BS) were measured. Un-decalcified frozen sections were also used for von Kossa staining.

### RNA analysis

The total RNA from flushed tibia were extracted using Trizol reagent (Invitrogen, Carlsbad, CA, USA). The Super-Script™ First-Strand Synthesis Kit (Takara Bio USA Inc, Mountain View, CA, USA) was used for the first-strand cDNA was synthetized. The iTaq™ Universal SYBR® Green Supermix kit (BIO‐RAD Laboratories Inc., Hercules, CA, USA) was used for real-time quantitative polymerase chain reaction (RT-qPCR). β-actin was used for normalization and calculation of relative mRNA level^37^. The primers sequence are listed in Table 1.

**Table 1:** Primers used for RT-qPCR.

Gene	Forward	Reverse
*β-Actin*	GTCGAGTCGCGTCCACC	CGCAGCGATATCGTCATCCA
*Alp*	GTGACTACCACTCGGGTGAAC	CTCTGGTGGCATCTCGTTATC
*Col1a1*	GGTCCTCGTGGTGCTGCT	ACCTTTGCCCCCTTCTTTG
*Runx2*	GTTCAACGATCTGAGATTTGTG	GGGAGGATTTGTGAAGACTG
*Ocn*	GAGGGCAATAAGGTAGTGAACAGA	AAGCCATACTGGTTTGATAGCTCG
*Dmp1*	CAACTGGCTTTTCTGTGGCAA	TGGGTGCGCTGATGTTTGCT
*Rankl*	CACCATCAGCTGAAGATAGT	CCAAGATCTCTAACATGACG
*Opg*	ATCCAAGACATTGACCTCTGTG	CTGTGGTGAGGTTCGAGTGG
*Gpr41*	TTCTGAGCGTGGCCTATCCA	AGACTACACTGACCAGACCAG
*Gpr43*	ATCCTCCTGCTTAATCTGACCC	CGCACACGATCTTTGGTAGGT
*Gpr109*	CCAAAAATGGCGAGGCATATCT	AAGAGGAACATAGCATCGTGC
*Igf1*	CACATCATGTCGTCTTCACACC	GGAAGCAACACTCATCCACAATG
*Eubacteria*	ACTCCTACGGGAGGCAGCAGT	ATTACCGCGGCTGCTGGC

### SCFA assay

SCFAs levels in cecal content of Ctrl and SCD mice receiving Ctrl or SCD feces were measured using gas chromatograph coupled to a mass spectrometer detector (GC–MS). Cecal content were collected from euthanized animals and immediately frozen to − 80 °C, then shipped to the Massachusetts Host-Microbiome Center at Brigham & Women's Hospital for measurement. Nine types of SCFAs were quantified: heptanoic acids, propionic, acetic, isobutyric, butyric, caproic, isovaleric, isocaproic, and valeric.

### Western blot analysis

Flushed tibia was used for protein extraction using 1 × radioimmunoprecipitation assay buffer (Cell Signaling). Same amount of protein was loaded on sodium dodecyl sulfate–polyacrylamide gel. After transfer, membrane was incubated for 1 h with 5% nonfat dry milk, then incubated at 4 °C for overnight with rabbit anti-IGF1 (Abcam) antibody, then incubated with anti-rabbit secondary antibody for 1 h at room temperature. Blots were developed with Super Signal West Dura Extended Duration Substrate (Thermo Scientific), then re-probed with actin antibody (Santa Cruz Biotechnology, Inc) for loading control.

### Serum measurements

At sacrificing, serum was collected from clotting blood. Serum IGF1 was measured by mouse/rat IGF1 Quantikine ELISA kit (R&D Systems, Inc) according to the manufacturer’s instructions.

### Statistical analyses

We used SPSS software for ANOVA analysis followed by Tukey for post hoc multiple comparisons. Statistical significance was defined as *p* < 0.05. All microbiome related statistics were performed using R version 4.1.0. Overall microbiome difference between Ctrl and SCD mice are tested by PERMANOVA and visualized by Non-metric Multi-dimensional Scaling (NMDS). Shannon diversity of microbiota was tested using wilcoxon *signed-*rank test. Specific taxa difference was tested using Deseq2. *p* values from multiple comparisons were adjusted by false discovery rate (FDR), and the results were considered significantly different at *Padj* < 0.05.

## Results

### Altered gut microbiota composition in SCD mice

To evaluate the gut microbiota changes, we collected fecal pellets from 3 month old Ctrl and SCD female mice (18–24 mice/group) and performed microbial community profiling. SCD and Ctrl mouse gut microbiome profiles were found to be significantly different (Fig. 1A). Microbiome diversity analysis showed alpha and beta diversity were significantly higher in SCD than Ctrl (Fig. 1B,C). At the Family level, fecal microbiota composition is markedly different between Ctrl and SCD mice (Fig. 1D). The relative abundance of family Bifidobacteriaceae and Erysipelotrichaceae are significantly increased in SCD mice compared to Ctrl mice. The relative abundance of family Bacteroidia-unclassified, Clostridiales-unclassified, Oscillospiraceae, Ruminococcaceae, and Muribaculaceae are significantly decreased in the SCD mice compared to Ctrl. The family Muribaculaceae (belong to the phylum Bacteroidetes, also referred to as S24-7, mouse intestinal bacteria, or Homeothermaceae^38^) was the highest relative abundant bacterial Family in Ctrl mice. These bacteria may be potential protective of bone loss in SCD since many bacteria under Family Muribaculaceae are implicated in the production of SCFA^38,39^.

### μCT showed impaired bone structural parameters of SCD mice was partially rescued after receiving FMT from Ctrl mice

**Figure 2:**
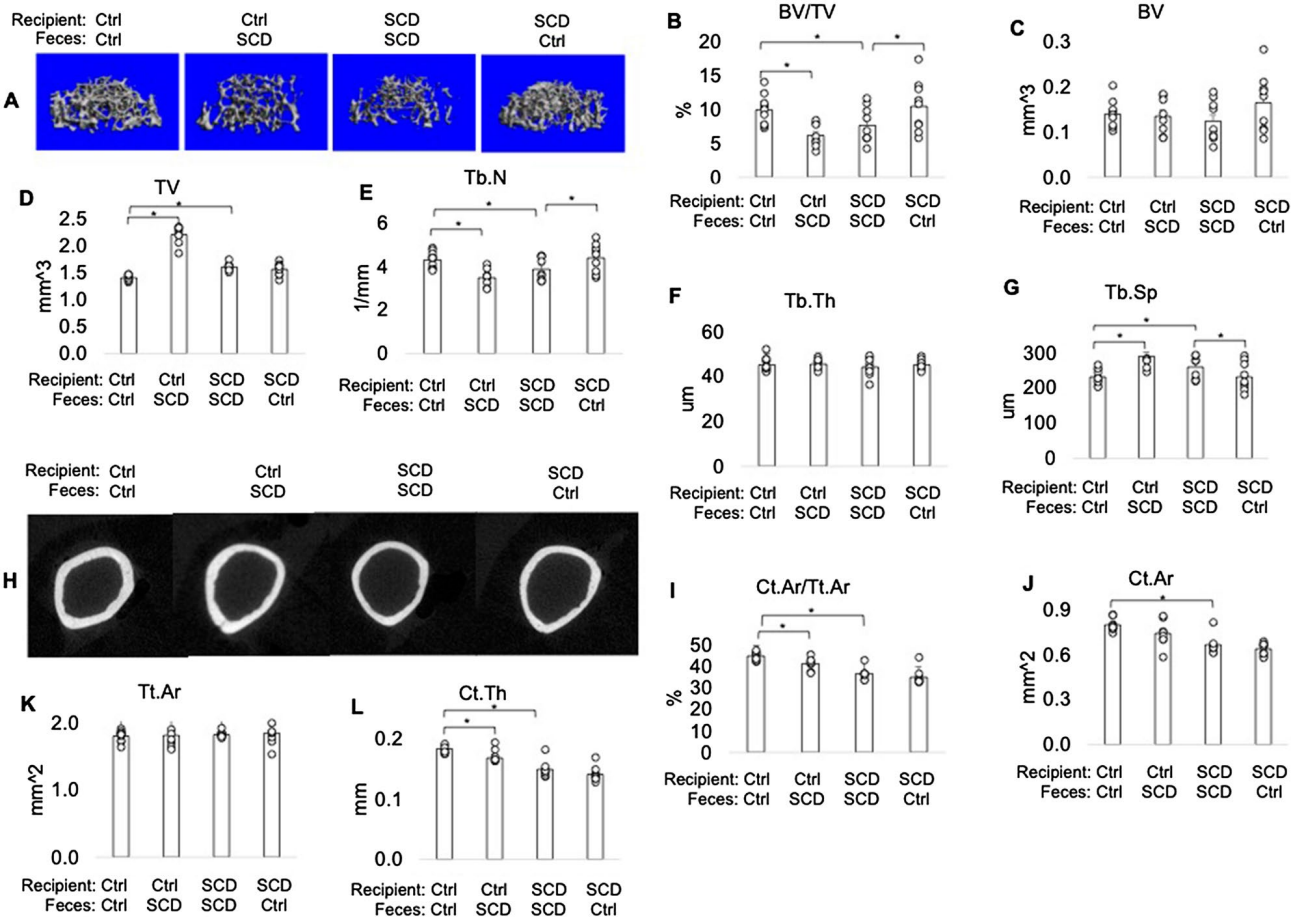
Transplantation of the gut microbiome from healthy Ctrl mice ameliorates bone loss in SCD female recipient mice. *μ*CT analysis of femur from Ctrl and SCD recipient female mice receiving feces from Ctrl or SCD female mice. Three-month-old Ctrl and SCD recipient mice subjected to FMT from Ctrl or SCD donor mice once a week. Six weeks-post FMT, samples were collected. (**A**) Representative μCT images of distal femur trabecular bone. (**B**) BV/TV, (**C**) BV, (**D**) TV, (**E**) Tb.N, (**F**) Tb.Th, and (**G**) Tb.Sp, of metaphysis. (**H**) Representative μCT images of mid-diaphysis cortical bone. (**I**) CtAr/TtAr, (**J**) Ct.Ar, (**K**) Tt.Ar, and (**L**) Ct.Th of mid-diaphysis. n = 9–10 mice/group. Data are shown as Mean ± SEM with individual data points. Statistical analyses were done using SPSS and *p* values were calculated using a two-way ANOVA with Tukey’s post hoc analysis. *p < 0.05.

**Figure 3:**
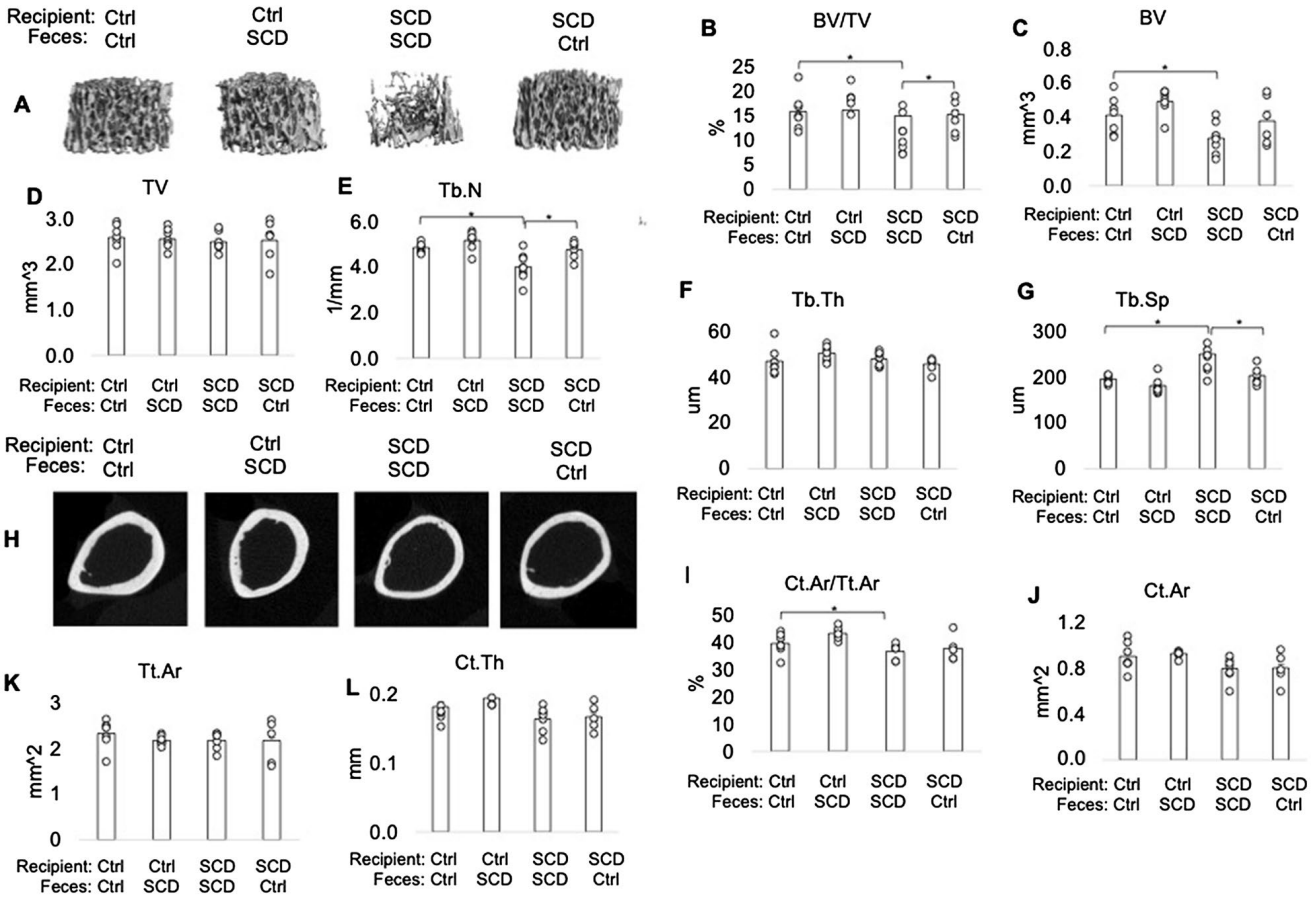
Transplantation of the gut microbiome from healthy Ctrl mice ameliorates bone loss in SCD male recipient mice. *μ*CT analysis of femur from Ctrl and SCD recipient male mice receiving feces from Ctrl or SCD male mice. Three-month-old Ctrl and SCD recipient mice subjected to FMT from Ctrl or SCD donor mice once a week. Six weeks-post FMT, samples were collected. (**A**) Representative μCT images of distal femur trabecular bone. (**B**) BV/TV, (**C**) BV, (**D**) TV, (**E**) Tb.N, (**F**) Tb.Th, and (**G**) Tb.Sp, of metaphysis. (**H**) Representative μCT images of mid-diaphysis cortical bone. (**I**) CtAr/TtAr, (**J**) Ct.Ar, (**K**) Tt.Ar, and (**L**) Ct.Th of mid-diaphysis. n = 6- mice/group. Data are shown as Mean ± SEM with individual data points. Statistical analyses were done using SPSS and *p* values were calculated using a two-way ANOVA with Tukey’s post hoc analysis. *p < 0.05.

FMT was performed from Ctrl to SCD to test if Ctrl microbiota can rescue SCD-like bone phenotypes and from SCD to Ctrl to test for a pathogenic effect of SCD microbiota on Ctrl mice (Fig. 1E). Microbiome diversity analysis showed that FMT of SCD feces to Ctrl mice increased alpha-diversity, but there was no change to beta-diversity compared to Ctrl mice before transplantation (Fig. 1F,G). FMT of Ctrl feces to SCD mice did not change to alpha-diversity, but decreased beta-diversity, compared to those before fecal transplant (Fig. 1H,I). To test if Ctrl microbiota can rescue SCD-like bone phenotypes, we performed uCT analysis in femurs at 6 weeks post FMT. μCT analysis was performed on both female (Fig. 2) and male (Fig. 3) mice. μCT analysis of femur from female mice (Fig. 2) showed the decreased bone volume/total volume (BV/TV) and trabecular number (Tb.N), as well as increased trabecular spacing (Tb.Sp) in SCD mice getting SCD feces compared with Ctrl mice getting Ctrl feces. FMT of Ctrl feces to SCD mice significantly increased BV/TV and Tb.N in SCD mice. In contrast, Tb.N and BV/TV_were_ decreased in Ctrl mice getting SCD feces compared with Ctrl mice getting Ctrl feces. Transplantation of Ctrl feces to SCD recipient did not alter decreased Ct.Ar, Ct.Ar/Tt.Ar, Tt.Ar in SCD mice. Decreased BV/TV and Tb.N in SCD male mice were also rescued after receiving fecal transplantation from Ctrl male mice (Fig. 3). We also performed μCT analysis on SCD only mice that did not receive either Ctrl or SCD feces and the data showed no difference in BV/TV between SCD only mice vs. SCD recipient receiving SCD feces (data not shown).

### Altered bone histomorphometry parameters of bone formation and resorption of SCD mice was partially ameliorated after getting FMT from Ctrl mice

**Figure 4:**
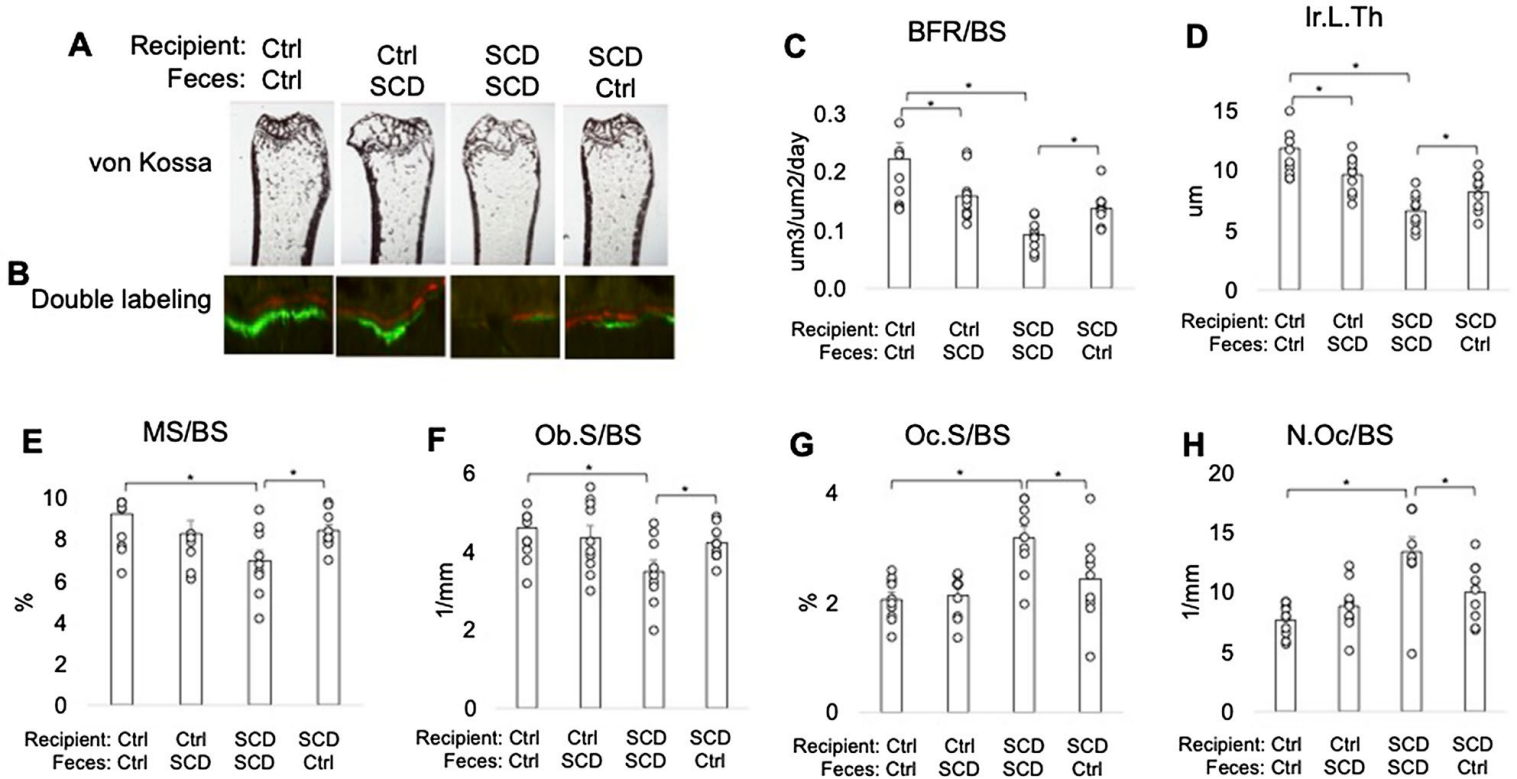
FMT of Ctrl feces rescued several bone phenotypes in SCD recipient mice. Bone histomorphometry. Three-month-old Ctrl and SCD female recipient mice were subjected to FMT from Ctrl or SCD donor mice once a week. Six weeks-post FMT samples were collected. (**A**) von Kossa staining shows decreased trabeculae in SCD mice receiving SCD feces compared with Ctrl mice receiving Ctrl feces that was partially rescued when SCD mice received Ctrl feces. Bone histomorphometry was conducted on metaphysis of isolated femur. (**B**) Calcein and Xylenol orange labeling shows decreased double-label distance in the SCD mice getting SCD feces compared to Ctrl mice getting Ctrl feces, which was partially rescued when SCD mice received Ctrl feces. (**C**) Bone formation rate/bone surface (BFR/BS), (**D**) inter-label thickness (Ir.L.Th), (**E**) mineral surface/bone surface (MS/BS), (**F**) OB surface per bone surface (ObS/BS), (**G**) OC number/bone surface (N.Oc/BS), (**H**) OC surface/bone surface (OcS/BS). n = 9–10 mice/group. Data are shown as Mean ± SEM with individual data points. Statistical analyses were done using SPSS and *p* values were calculated using a two-way ANOVA with Tukey’s post hoc analysis. *p < 0.05.

To determine the effects of FMT on bone at structure level, bone histology was conducted on femurs collected at the end of the experiment. Von Kossa staining revealed decreased trabeculae in SCD mice getting SCD feces compared with Ctrl mice getting Ctrl feces that that was partially rescued in SCD mice when receive Ctrl feces (Fig. 4A). To examine osteoblast and osteoclast function at the cellular level bone histomorphometry was performed. Dynamic histomorphometry showed decreased Ir.L.Th, MS/BS, and BFR/BS in the SCD mice fed SCD feces compared with Ctrl mice fed Ctrl feces, and these parameters were partially rescued in SCD mice fed Ctrl feces (Fig. 4B–E). Osteoblast surface/bone surface (OS/BS) was lower in SCD mice fed SCD feces compared with Ctrl mice fed Ctrl feces, and were partially rescued with FMT from Ctrl feces (Fig. 4F). Osteoclast surface/bone surface (Oc.S/BS) and osteoclast number/bone surface (N.Oc/BS) were higher in SCD recipient receiving SCD feces compared with Ctrl recipient receiving Ctrl feces, and were partially rescued with FMT from Ctrl feces (Fig. 4G,H). These data suggest that FMT from Ctrl feces significantly reduced the abnormalities in histomorphometry indices in SCD mice.

### Altered OB and OC-related gene expression in SCD mice was partially rescued after receiving FMT from Ctrl mice

**Figure 5:**
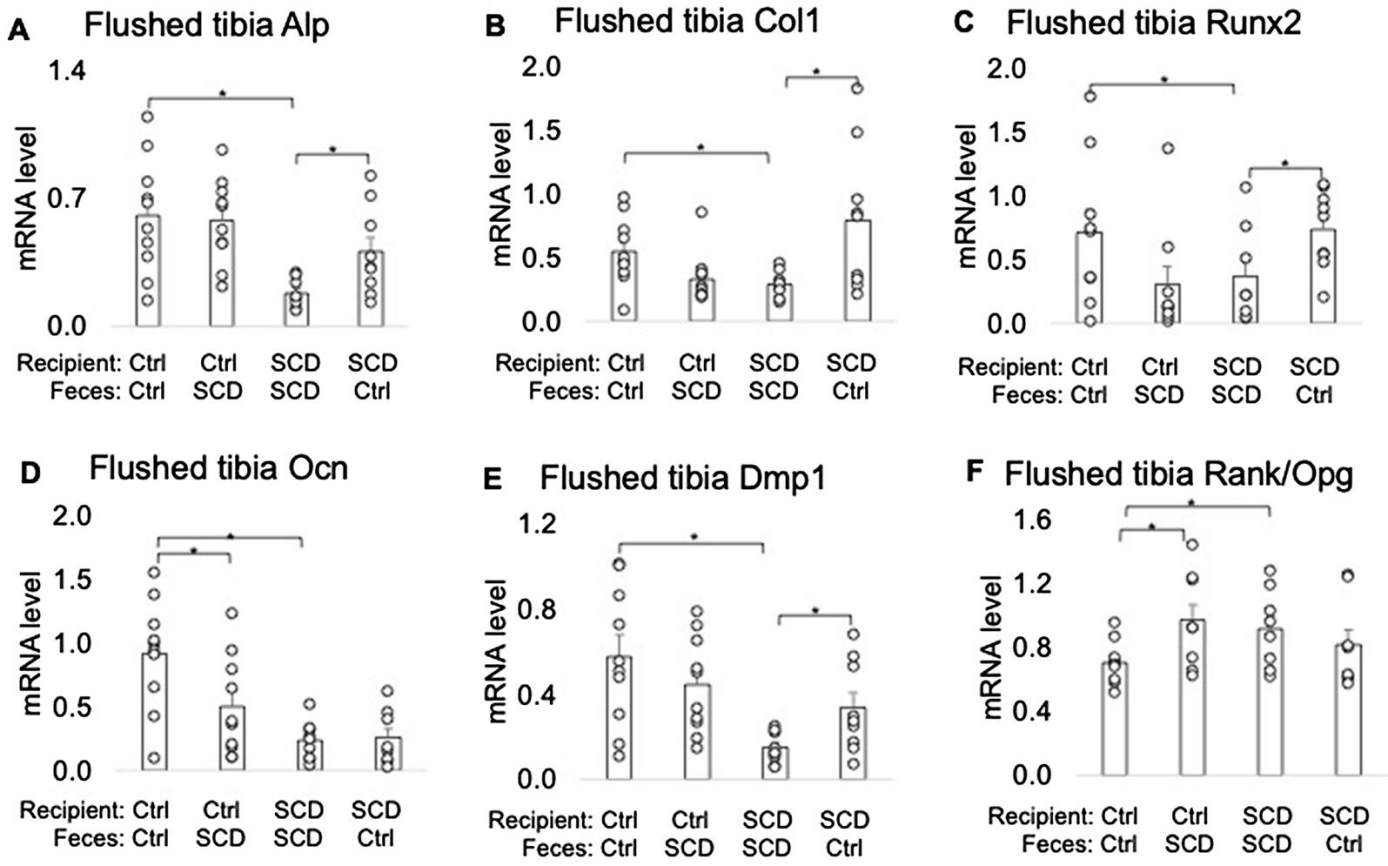
Effect of FMT on level of bone‐marker genes in flushed tibias from Ctrl and SCD recipients. Three-month-old Ctrl and SCD female recipient mice subjected to FMT from Ctrl or SCD donor mice once a week. Six weeks post FMT samples were collected. Flushed tibia were used for RNA isolation. Quantitative real‐time PCR analysis was conducted for the following gene expression: (**A**) *Alp*, (**B**) *Col1*, (**C**) *Runx2*, (**D**) *Ocn*, (**E**) *Dmp1*, and (**F**) *Rankl/Opg*. n = 9–10 mice/group. Data are shown as Mean ± SEM with individual data points. Statistical analyses were done using SPSS and *p* values were calculated using a two-way ANOVA with Tukey’s post hoc analysis. *p < 0.05.

We assessed whether improved bone formation in SCD mice receiving feces of Ctrl mice was related to changes in OB formation and OC differentiation genes. Quantitative PCR of flushed tibiae mRNA revealed that bone formation marker gene Alp, type-1 collagen (Col1), Osteocalcin (Ocn), and Dmp1 mRNA were significantly decreased in tibiae shaft from SCD mice getting SCD feces vs. Ctrl mice getting Ctrl feces. Bone resorbing marker gene Rankl/Opg ratio was significantly increased in SCD mice fed SCD feces vs. Ctrl mice fed Ctrl feces. Transplantation of Ctrl feces to SCD mice significantly increased expression of Alp, Col1, Runx2, and Dmp1 in SCD mice. FMT of SCD feces to Ctrl mice significantly decreased expression of Col1, Runx2, Ocn, and increased Rankl/Opg in Ctrl mice (Fig. 5). These results suggest that the gut microbiota is a mediator of SCD-induced bone loss and gut microbiota from healthy Ctrl mice may be protective for bone loss in SCD mice.

### Transplantation of Ctrl feces improved decreased cecal SCFA in SCD recipient

**Figure 6:**
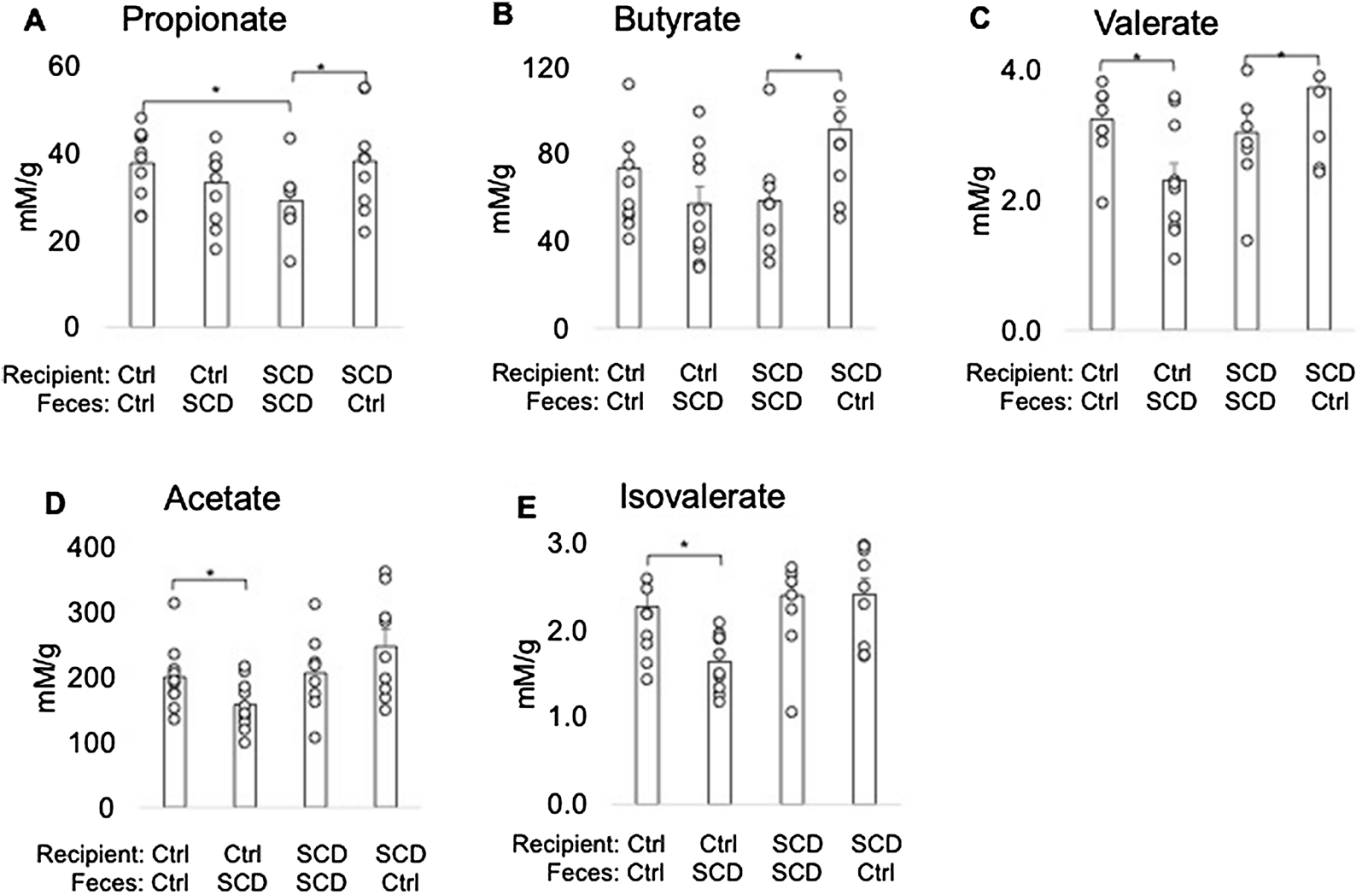
Cecal SCFA levels in Ctrl and SCD recipient mice receiving feces from Ctrl or SCD mice. Three-month-old Ctrl and SCD female recipient mice subjected to FMT from Ctrl or SCD donor mice once a week. Six weeks post-FMT, cecal contents were collected and SCFA levels were measured by GC–MS. (**A**) Propionate, (**B**) Butyrate, (**C**) Valerate, (**D**) Acetate, and (**E**) Isovalerate. n = 9–10 mice/group. Data are shown as Mean ± SEM with individual data points. Statistical analyses were done using SPSS and *p* values were calculated using a two-way ANOVA with Tukey’s post hoc analysis. *p < 0.05.

The relative abundance of Muribaculaceae family is significantly decreased in the SCD mice compared with Ctrl. Many of these bacteria that are implicated are SCFA producers^38,39^. To determine if FMT alter SCFA production we measured SCFAs level in cecal content. As shown Fig. 6, there is decreased cecal propionate in SCD mice fed SCD feces vs. Ctrl mice fed Ctrl feces. Transplantation of Ctrl feces to SCD recipient significantly increased cecal butyrate, propionate, and valerate level. In contrast, feeding SCD feces to Ctrl recipient significantly decreased acetate and isovalerate level.

### Transplantation of Ctrl feces improved decreased Gpr41 and Gpr43 mRNA level in flushed tibia of SCD mice

**Figure 7:**
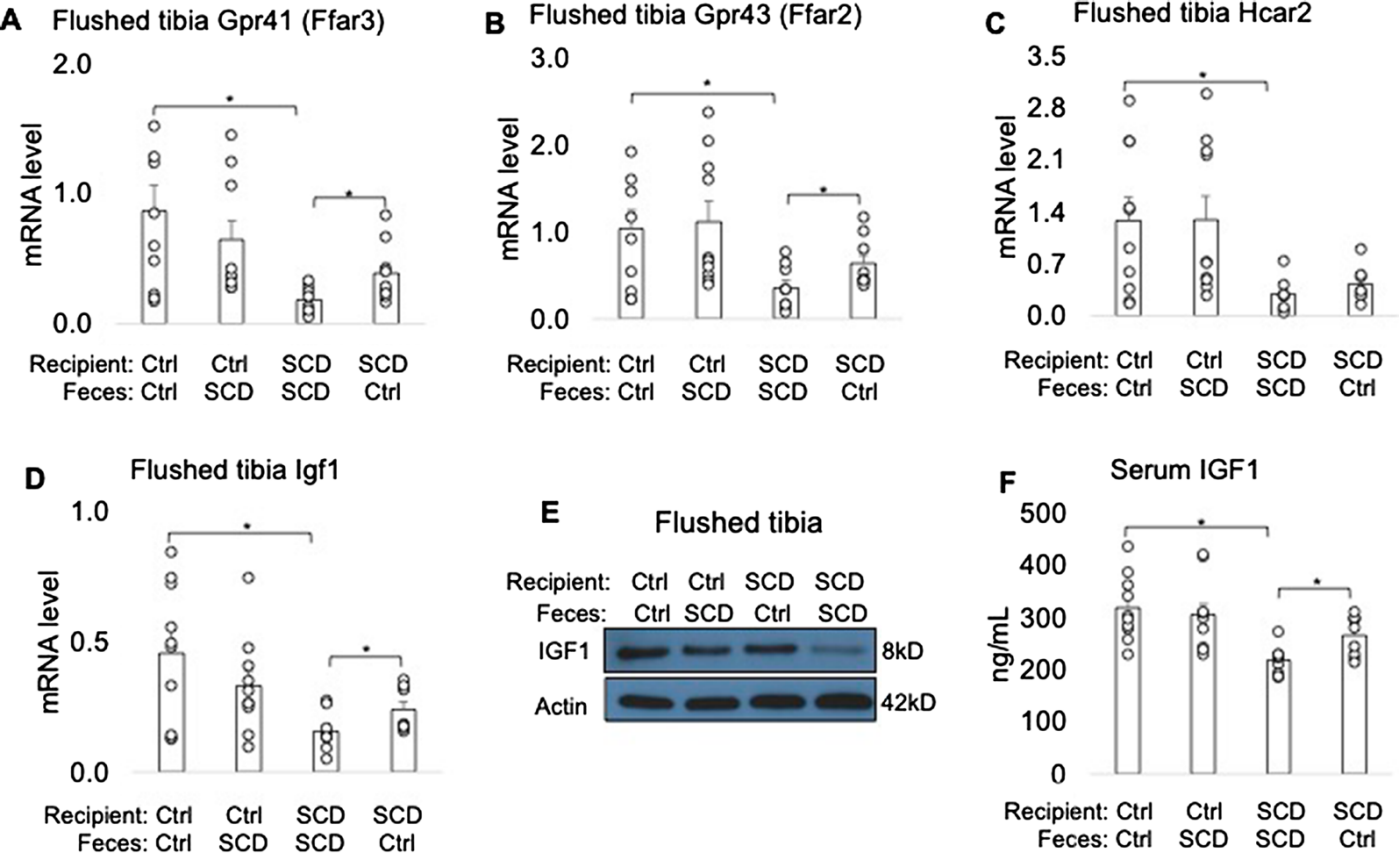
FMT from Ctrl to SCD restored *Gpr41 and Gpr43* mRNA levels in flushed tibia, and IGF1 level in bone and serum. Three-month-old Ctrl and SCD female mice subjected to FMT from Ctrl or SCD donor mice once a week. Six weeks post FMT samples were collected. qPCR analysis of flushed tibia for (**A**) *Gpr41*, (**B**) *Gpr43*, (**C**) *Gpr109, and* (**D**) *Igf1* mRNA levels. n = 9–10 mice/group. (**E**) IGF1 protein in flushed tibia was determined by western blot. Samples are pooled from 9–10 mice per group. Uncropped Western blots are shown in Supplemental Fig. 2. (**F**) IGF1 level in serum measured by ELISA. n = 9–10 mice/group. Data are shown as Mean ± SEM with individual data points. Statistical analyses were done using SPSS and *p* values were calculated using a two-way ANOVA with Tukey’s post hoc analysis. *p < 0.05.

Since G protein-coupled receptor 41 (GPR 41), GPR43, and GPR109 have been identified as endogenous receptors for SCFAs^40–43^ and have been found in both osteoclasts and osteoblasts^44^, we measured their mRNA levels in flushed tibia. As shown in Fig. 7A–C, 41 (GPR 41), GPR43, and GPR109 mRNA level were significantly lower in SCD recipient receiving SCD feces compared to Ctrl recipient receiving Ctrl feces. Transplantation of Ctrl feces to SCD recipient significantly improved *Gpr41* and *Gpr43* mRNA level in SCD mice.

### FMT of Ctrl feces improved IGF1 level in bone and serum of SCD recipient mice

There is decreased IGF1 level in both SCD patients^28^ and mice^29^. Because we found the gut microbiota dysbiosis-mediated decrease in SCFA (Fig. 6) and it is reported that SCFA regulates IGF1 production^21^, we examined whether transplantation of Ctrl feces could rescue the decreased IGF1 in SCD female mice. As shown in Fig. 7D–F, we found significantly decreased IGF1 mRNA and protein level in flushed tibia and serum in SCD mice fed SCD feces compared with Ctrl mice fed Ctrl feces, whereas transplantation of Ctrl feces to SCD recipient restored IGF1 level in bone to Ctrl mice levels.

## Discussion

This study demonstrated a striking change in the gut microbiota composition. The relative abundance of Muribaculaceae family is significantly decreased in the SCD mice compared with Ctrl. Our findings are the first to identify the gut microbiota as mediators of sickle cell bone loss. We are also the first to report decreased SCFA in SCD mice. Specifically, modifying the microbiota (FMT, to alter the microbiota toward a beneficial balance) significantly blunted SCD-induced bone loss. We also demonstrate that FMT from healthy Ctrl feces improved decreased cecal SCFA and their receptors GPR41/43 in bone, with concomitant improvement on IGF1 in serum and bone and improved osteoblast functions in SCD mice.

In this study, we found that family Muribaculaceae was the highest relative abundant bacterial Family in Ctrl mice. The relative abundance of family Muribaculaceae is significantly decreased in the SCD mice compared with Ctrl. These bacteria together accounted for a large proportion of the total gut bacteria, 45% in Ctrl, and 25% in SCD and they are the most significantly decreased bacteria family in SCD. These bacteria may be potentially protective of bone loss in SCD since: (1) many bacteria under family Muribaculaceae are implicated in the production of SCFA^38,39^; (2) the taxon belongs to Muribaculaceae positively covary with intestinal barrier function^45^ and has significant positive correlation with intestinal tight junction protein level^46^; (3) Muribaculaceae also showed a negative association with pro-inflammatory cytokine level^46^. Further studies are needed to identify bacterial strain(s) that could be used as probiotics in SCD bone loss.

Mice were treated with laxative PEG before FMT. This is a widely used, economic approach to largely deplete the gut microbiome in recipient mice. Treating mice with a cocktail of antibiotics is another way to deplete the gut microbiota to enhance FMT efficiency. However, antibiotic treatment could have multiple effects, including decreasing aged neutrophils^18^ that we have shown to impact osteoblast function in SCD mice^47^. We believe that PEG treatment is a more physiologically relevant model for our studies. The PEG treatment is easy to use for diminishing gut resident bacteria. In contrast to antibiotics, PEG temporarily decrease the abundance of gut microbiota in mice. Bowel cleansing also kept indigenous microbiota after-treatment compared to antibiotics^33,48^. We found Ctrl mice received microbiota derived from SCD mice had an increased alpha diversity, compared to these mice before transplantation. This is consistent with higher alpha diversity we observed in SCD mice at the baseline. But beta-diversity were similar in Ctrl mice before and after transplantation. In SCD recipient, alpha diversity was statistically similar as SCD mice before transplantation of microbiota derived from Ctrl. However, we found SCD recipient mice had a decreased beta-diversity, compared to those before fecal transplant, suggesting transplantation reduces inter-subject variation of SCD mice. This is also consistent with lower beta-diversity in Ctrl mice at baseline. These findings suggest FMT successfully transferred alpha or beta diversity phenotypes in the donor stools. Studies by others have shown that SCFA affect bone metabolism^49^. However, to date there are no studies reporting on the SCFA level or addressing the role of SCFA in SCD bone disease. Here we used FMT approach and found that gut microbiota production of SCFA is an important mediator in determining bone mass in SCD mice. Thus, SCFA linked gut microbiota and bone homeostasis in SCD.

It is reported that SCFA affect systemic and local immune functions, and inhibit bone resorption and osteoclast differentiation in vivo and vitro^49^. Consistent with this we found that FMT from healthy Ctrl feces rescued the increased osteoclast number in SCD mice, and rescued the increased intestinal inflammatory cytokine in SCD mice (data not shown). The SCFA level in the bone marrow is high enough to directly inhibit in vivo osteoclast differentiation^49^. GPR41, GPR43, and GPR109 have been found as endogenous receptors for SCFAs^40–43^. SCFAs can direct act on cells by activating GPRs^50^. Furthermore, the SCFA butyrate and propionate may have GPR-independent effects by acting as histone deacetylase inhibitors^51^. Although osteoclasts precursors express the receptors for SCFA, and propionate and butyrate can inhibit osteoclast differentiation directly^52^, it has been reported that the suppressive capacity of acetate, propionate and butyrate on osteoclast differentiation and bone resorption was independent of the GPR41, GPR43^49^, and GPR109^52^. Therefore, FMT-mediated SCFA reduction in SCD mice may directly active osteoclast through histone deacetylase in SCD mice. Further studies using osteoclast-specific GPRs null SCD mice to clarify the direct effect of SCFA on osteoclast in SCD are needed.

IGF1 is a growth factor with both endocrine and paracrine/autocrine actions on bone^21,53–55^. In this study we found a significant improvement on osteoblast number, osteoblast activity, and bone formation related genes in SCD mice after receiving Ctrl feces, and these are associated with improvement on SCFA level and IGF1 production. Thus, increased IGF1 could contribute to improved bone formation in SCD mice receiving Ctrl feces. It is reported that gut microbiota and sodium butyrate can affect osteoblast precursors^56,57^. Since we found the concomitant changes in SCFA, GPR41, GPR43 and IGF1 in SCD mice after receiving FMT from Ctrl feces, it is possible that decrease of SCFAs leads to less GPR41/43 expression in bone, and subsequently reduce IGF1 production are responsible for low bone mass in SCD mice. Osteoblast-specific GPR41/43 null mice will be utilized to probe the involvement of these receptors in the signaling axis leading from gut microbiome alterations to bone changes in SCD in future studies.

Results from animal and human studies on sex differences in gut microbiota are inconsistent, some showed sex-related differences in gut microbiota and some studies showed no such sex difference^58^. Although both genders are used from animal and human SCD microbiota studies by other groups^17,59^, the sex-related gut microbiota differences are not reported. In clinic the male SCD is twice more likely to develop sickle cell complications than the female patients partially due to sex hormone regulated nitric oxide (NO) production^60^. It is known that microbiota regulates NO^61^. In the current study, 16S rRNA sequencing was only performed in female mice, therefore, the magnitude of the contribution of sex to the gut microbiota-regulated bone change is not clear in SCD which is a limitation of the study.

In summary, in this study we demonstrated that microbial dysbiosis contributes to bone pathogenesis in SCD mice. Healthy gut microbiota community of Ctrl mice is protective for SCD-related bone loss by increasing the level of bone growth factor IGF1 in response to increased bacterial metabolites SCFAs. Therapeutic supplementation of SCFA or SCFA-producing probiotic/prebiotic may provide a powerful instrument to prevent bone loss in SCD patients by increasing the endogenous level of SCFA.

## Supplementary Information

Supplementary Information.

## Data Availability

The datasets generated and analyzed during the current study are available in the NCBI repository, Accession Number: PRJNA853784.
