# Supplementary material for: Bone loss is ameliorated by fecal microbiota transplantation through SCFA/GPR41/ IGF1 pathway in sickle cell disease mice
Source: Sci Rep. 2022 Nov 30;12:20638. doi: 10.1038/s41598-022-25244-9 (PMC9712597; doi:10.1038/s41598-022-25244-9)
Supplement: Supplementary file 1 — Supplementary Information. [file 41598_2022_25244_MOESM1_ESM.pdf]

Supplemental Figures

Bone Loss is Ameliorated by Fecal Microbiota Transplantation through SCFA/GPR41/ IGF1 Pathway in Sickle Cell Disease Mice

Liping Xiao[1] *, Yanjiao Zhou[1], Suresh Bokoliya[1], Qingqi Lin[1], Marja Hurley[1]

[1]Department of Medicine, School of Medicine, UConn Health, Farmington, CT, 06030, USA

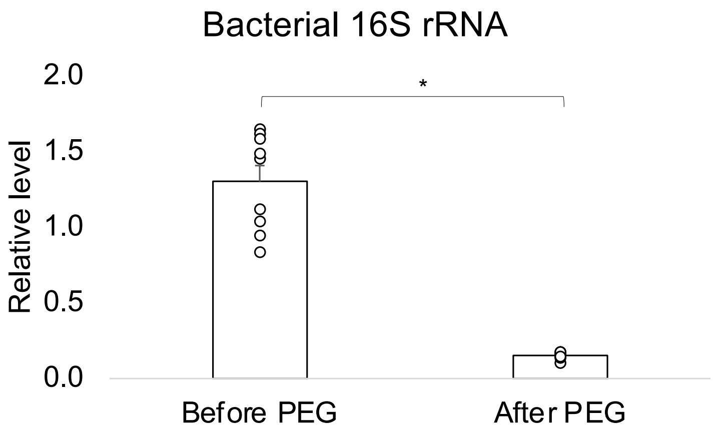

Supplemental Figure 1. Decreased bacterial load after PEG treatment in Ctrl mice. n=9 mice/group. Data are Mean±SE. *p<0.05 by T test.

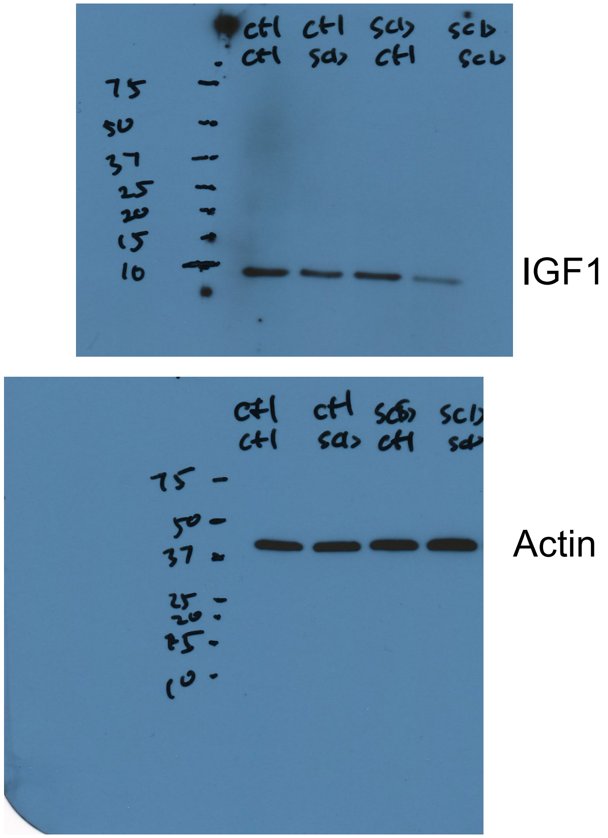

Supplemental Figure 2. Uncropped Western blots for Figure 7E.
